# Jamming state transition and collective cell migration

**DOI:** 10.1186/s13036-019-0201-4

**Published:** 2019-09-05

**Authors:** Ivana Pajic-Lijakovic, Milan Milivojevic

**Affiliations:** Faculty of Technology and Metallurgy, Belgrade University, Karnegijeva 4, Belgrade, Serbia

**Keywords:** Long-time cell rearrangement, Viscoelasticity of multicellular surfaces, Tissue surface tension, Jamming state transition, Collective cell migration

## Abstract

Jamming state transition has been used in literature to describe migrating-to-resting cell state transition during collective cell migration without proper rheological confirmation. Yield stress often has been used as an indicator of a jamming state. Yield stress points to the liquid-to-solid state transition, but not a priori to jamming state transition. Various solid states such as elastic solid and viscoelastic solids can be considered in the context of their ability to relax. The relaxation time for (1) an elastic solid tends to zero, (2) Kelvin-Voigt viscoelastic solid is finite, and (3) jamming state tends to infinity.

In order to clarify the meaning of jamming state from the rheological standpoint we formulated the constitutive model of this state based on following conditions (1) migration of the system constituents is much damped such that the diffusion coefficient tends to zero, (2) relaxation time tends to infinity, (3) storage and loss moduli satisfy the condition *G*^′^(*ω*)/*G*^"^(*ω*) = *const* > 1. Jamming state represents the non-linear viscoelastic solid state. The main characteristic of this state is that the system cannot relax.

Jamming state transition of multicellular systems caused by collective cell migration is discussed on a model system such as cell aggregate rounding after uni-axial compression between parallel plates based on the data from the literature. Cell aggregate rounding occurs via successive relaxation cycles. Every cycle corresponds to a different scenario of cell migration. Three scenarios were established depending on the magnitude of mechanical and biochemical perturbations (1) ordered scenario with reduced perturbations corresponds to the case that most of the cells migrate, (2) disordered scenario corresponds to the case that some cell groups migrate while the others (at the same time) stay in resting state (corresponds to medium perturbations), and (3) highly suppressed cell migration under large perturbations corresponds to the viscoelastic solid under jamming state. If cells reach the jamming state in one cycle, they are able to overcome this undesirable state and start migrating again in the next cycle by achieving the first or second scenarios again.

## Background

Main features of cell rearrangement during collective cell migration related to the viscoelasticity of multicellular surfaces are important for the deeper understanding of various biological processes such as wound healing, tumorigenesis, and morphogenesis [1–6]. Various multicellular surfaces have been considered under in vivo and in vitro conditions. Mikami et al. [3] discussed collective cell migration of the multicellular surfaces in the form of stratified epithelial cells toward the wounds. A number of sheets and their sizes depend on the size, shape and depth of injury. Pajic-Lijakovic and Milivojevic [7–9] considered the rearrangement of the multicellular surface region during cell aggregate rounding after uniaxial compression between parallel plates. Guevorkian et al. [10] considered the rearrangement of the aggregate surface part under micropipette aspiration. Long-time viscoelasticity of multicellular surfaces depends on: (1) the configuration of migrating cells and the rate of its change, (2) the volume fraction of migrating cells, (3) the viscoelasticity of migrating cell groups, and (4) the viscoelasticity of surrounding resting cells [7, 8]. Configuration changes occur via local migrating-to-resting cell state transitions and vice versa. These transitions have been considered as jamming state transitions [11–16]. Garcia et al. [11] indicated parameters that influence cell jamming (1) cellular packing density which depends on cell type and growth conditions, (2) cell−cell adhesion energy, (3) magnitude of cellular forces and persistence time for these forces, and (4) cell shape. They pointed out that cell monolayers behave as amorphous solids under reduced cell velocities. Sharp phase transitions didn’t observe, rather a dynamic change in the dominant internal forces that control the motion.

Jamming state transition is a rheological term which represents transition from liquid-like state to solid-like state [17–20]. Jamming state, similarly as glass state corresponds to a much damped movement of the system constituents. This damped state could be induced by an increase of packing density (the jamming state transition) or by a decrease of temperature (the glass state transition). Many physical systems such as granular systems, polymer hydrogels and various types of multi-phase systems show similar behavior. Unjamming-to-jamming state transition and jamming state itself for viscoelastic systems have been characterized rheologically [17–20]. Tighe [17] and Braumgarten and Tighe [19] reported that systems have to pass through a transition regime before reaching the jamming state. The main characteristic of the transition regime is (1) storage modulus is equal to loss modulus, i.e. *G*^′^(*ω*) = *G*^"^(*ω*) and (2) scaling exponent for storage and loss moduli vs. angular velocity is equal to 1/2 . Storage modulus quantifies storage energy, while the loss modulus quantifies energy dissipation. Honter and Weeks [18] characterized rheologically the jamming/glass state of colloidal systems such that (1) storage modulus is higher than loss modulus, i.e. *G*^′^(*ω*)/*G*^"^(*ω*) > 1, (2) diffusion coefficient of the system constituents tends to zero, i.e. *D* → 0, and (3) the system relaxation time tends to infinity, i.e. *τ*_*R*_ → ∞. Consequently, a system under jamming state cannot relax. The aim of this consideration is to formulate a constitutive model for describing the jamming state and to discuss various viscoelastic states from the standpoint of rheology. This is a prerequisite of deeper understanding of the jamming state transition in multicellular systems caused by collective cell migration.

Mongera et al. [21] recently used yield stress as the main indicator of the jamming state transition during collective cell migration. Yield stress could be an indicator of reaching the solid state. However, there are many various solid states such as elastic solid or linear and non-linear viscoelastic solids while the jamming state is just one of them. Various solid states are characterized by various constitutive models and corresponding relaxation times. Linear viscoelastic solid can be described by various constitutive models such as Kelvin-Voigt model, Zener model, Burgers model and multi-parameter models. Kelvin-Voigt model is the simplest linear model which describes constitutive behavior of viscoelastic solid. Corresponding relaxation time is finite *τ*_*R*_ > 0. The relaxation time for the elastic solid is *τ*_*R*_ = 0. However, jamming state satisfies the condition that the relaxation time tends to infinity *τ*_*R*_ → ∞ as reported by Honter and Weeks [16].

For further consideration, it is necessary to discuss migrating-to-resting cell state transition in the context of liquid-to-solid state transition. Some authors have described migrating cells as a viscoelastic liquid [13, 15, 22, 23]. Lee and Wolgemuth [23] considered collective cell migration within 2D cell monolayers and proposed Maxwell model suitable for viscoelastic liquid, but without rheological confirmation. Flenner et al. [22] treated cell aggregate rounding caused by collective cell migration as a viscoelastic liquid. Flenner et al. [22] and Oswald et al. [15] introduced two inter-connected claims (1) cell aggregate rounding is driven by surface tension and (2) the surface tension represents the characteristic of the liquid. We agree that aggregate rounding is driven by tissue surface tension. However, surface tension is not necessarily the characteristic of the liquid. Amorphous viscoelastic solids such as polymer hydrogels and foams also have surface tension [24]. Pajic-Lijakovic and Milivojevic [25] pointed that cell aggregate rounding after uni-axial compression leads to (1) shape relaxation from ellipsoidal to spherical, (2) surface decrease from initial to the equilibrium value, and (3) surface strain relaxation. Strain relaxation ability is the characteristic of viscoelastic solid rather than viscoelastic liquid. Doxzen et al. [26] reported that migrating cell groups behave as rigid bodies. All cells within the migrating group move, maintaining cell-cell adhesions. Internal mechanical effects within the groups have been described by plithotaxis [1]. Plithotaxis represents collective cell guidance by cooperative intracellular forces which ensure the integrity of the groups. Migrating-to-resting cell state transition induces a change in the state of viscoelasticity. However, it hasn’t been confirmed rheologically that this “resting” state is a priori the jamming state. Few types of 2D and 3D multicellular systems have been discussed in the literature in the context of jamming state transition such as (1) cell monolayers during expansion [13, 14, 16], (2) cell aggregate rounding after uni-axial compression between parallel plates [15], and (3) cell sorting into different homogeneous domains with one cell type becoming engulfed by the other [15]. Bi et al. [13] considered cell monolayers and developed the vertex model to describe mechanical energy of a single cell under simplified conditions such as homogeneous, isotropic confluent tissue monolayers at constant density as the sum of three types of contributions (1) cell bulk elasticity, (2) cell contractility, and (3) interfacial energy. However, multicellular systems are inhomogeneous and anisotropic even in 2D. Notbohm et al. [27] and Nnetu et al. [28] experimentally obtained that cell monolayers represent highly perturbed non-homogeneous structures.

The main goal of this consideration is to describe migrating-to-resting cell state transition from the standpoint of rheology and discuss the possibility of (1) determining the jamming state in multicellular systems and (2) cells to overcome this suppressed state and start moving again. The rheological consideration of long-time cell rearrangement was given on simplified multicellular systems such as 3D cell aggregates rounding after uni-axial compression under in vitro conditions based on experimental data proposed in the literature.

## Theoretical background

Jamming state transition has been related to the liquid-to-solid state transition in the literature. The jamming state is the rheological term and needs additional characterization. Many amorphous viscoelastic systems such as granular systems, polymer hydrogels and various types of multi-phase systems show jamming/glass state transition. Jamming state transition is caused by a packing density increase while glass state transition is caused by a temperature decrease. We will discuss the main characteristics of the jamming/glass state transition from the rheological standpoint.

### Various viscoelastic states

We consider and compare various types of viscoelastic behavior in the context of (1) liquid-to-solid state transition and vice versa and (2) relaxation ability in order to clarify jamming/glass state from the rheological standpoint. Considered types of viscoelasticity are (1) viscoelastic liquid described by Maxwell model, (2) viscoelastic solid described by Kelvin-Voigt model, (3) transition regime as the prerequisite for jamming/glass state transition [17, 19], and (4) jamming/glass state [16].

The Maxwell model equation is the simplest linear model suitable for describing viscoelastic liquid behavior. It is expressed as:

1 $$ \sigma (t)+{\tau}_{R1}\overset{.}{\sigma }(t)=\eta \overset{.}{\varepsilon }(t) $$

where *σ*(*t*) is the stress, *ε*(*t*) is the strain, $\overset{.}{\varepsilon }(t)=\frac{d\varepsilon (t)}{d t}$ is strain rate, *τ*_*R*1_ is the stress relaxation time, and 휼 is the viscosity. Strain change under constant stress condition *σ*_0_ for the initial condition *ε*(0) = 0 is equal to $\varepsilon\ (t)=\frac{\sigma_0}{\eta }t$. Strain increases during the time period *∆t* from *ε*(0) = 0 to *ε*(*∆t*) without the ability to relax. If the system undergoes free relaxation at *t* = *∆t* such that *σ* = 0 and *ε*(*∆t*) = *ε*_0_, the strain stays constant for *t* > *∆t* and equal to *ε*(*t*) = *ε*_0_. Consequently, a strain cannot relax under constant stress condition due to sample fluidity. It is the main characteristic of a viscoelastic liquid. Stress relaxation under constant strain rate ${\dot{\varepsilon}}_0$ could be expressed starting from the initial condition *σ*(*t* = 0) = 0 as $\sigma (t)={\sigma}_R\left(1-{e}^{-\frac{t}{\tau_{R1}}}\right)$ (where *σ*_*R*_ is the residual stress equal to ${\sigma}_R=\eta\ {\overset{.}{\varepsilon}}_0$).

Equation 1 could be transformed from the time domain into the frequency domain using the Fourier integral transform. Transforming equation is expressed in form *F*[*σ*(*t*)] = *G*^∗^(*ω*)*F*[*ε*(*t*)] (where *F*[∙] is the Fourier transform, *ω* is the angular velocity, and *G*^∗^(*ω*) is the complex modulus). The complex modulus is equal to:

2 $$ {G}^{\ast}\left(\omega \right)={G}^{\prime}\left(\omega \right)+i\ G"\left(\omega \right) $$

where *G*^′^(*ω*) is the storage modulus, *G* " (*ω*) is the loss modulus, and $i=\sqrt{-1}$ is the imaginary unit. The storage modulus *G*^′^(*ω*) quantifies elastic behavior while the loss modulus *G* " (*ω*) quantifies viscous behavior of the examined system. Storage and loss moduli could be expressed from eqs. 1 and 2:

3 $$ {G}^{\prime}\left(\omega \right)=\frac{{\upeta \tau}_{R1}{\omega}^2}{1+{\tau}_{R1}^2{\omega}^2}\ {G}^{"}\left(\omega \right)=\frac{\upeta \upomega}{1+{\tau}_{R1}^2{\omega}^2} $$

**Fig. 1:**
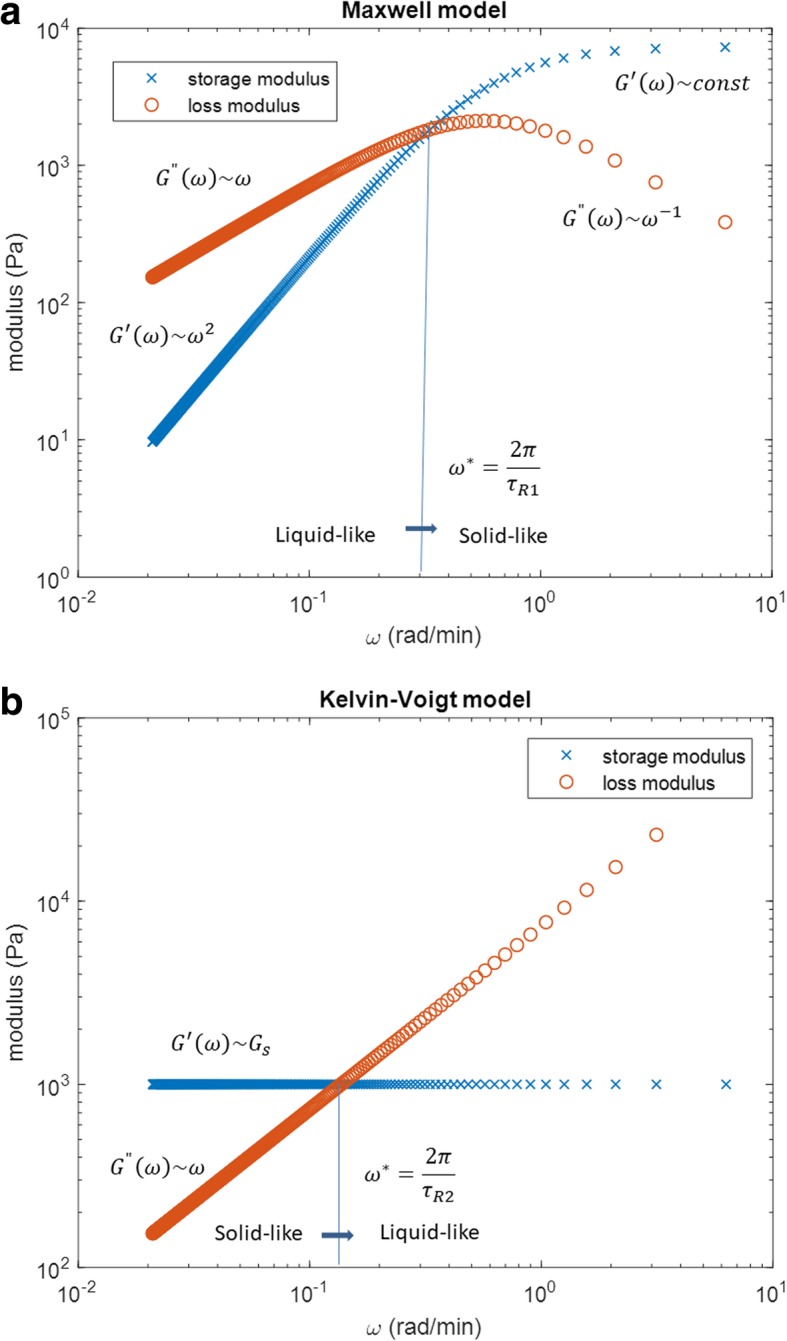
Storage and loss moduli vs. angular velocity for: **a** The Maxwell model calculated by eq. 3 for the model parameters: the relaxation time for stress *τ*_*R*1_ = 3 *min* and the viscosity η = 7.3x10^3^*Pa min*; (This relaxation time corresponds to the order of magnitude of stress relaxation time for cell aggregate uniaxial compression between parallel plates while the viscosity corresponds to the value obtained for epithelial cell aggregate obtained by Marmottant et al. [29].) **b** The Kelvin-Voigt model calculated by eq. 5 for the model parameters: the elastic modulus *G*_*s*_ = 1 *kPa* and the viscosity η = 7.3x10^3^*Pa min*. (The value of the elastic modulus is in the range of the experimental values obtained for soft tissue [30])

Storage and loss moduli satisfy the following conditions (1) *G*^′^(*ω*)/*G*^"^(*ω*) < 1 at low angular velocities (liquid-like behavior), (2) *G*^′^(*ω*)/*G*^"^(*ω*) > 1 at high angular velocities (solid-like behavior), (3) *G*^′^(*ω*)~*ω*^2^ and *G*^"^(*ω*)~*ω* at low angular velocities and (4) *G*^′^(*ω*)~*const* and *G*^"^(*ω*)~*ω*^−1^ at high angular velocities. Stress relaxation time could be obtained on two ways (1) by comparing experimental data of stress vs. time with model prediction obtained by eq. 2 or (2) by comparing experimental data of storage and loss moduli vs. angular velocity with model prediction obtained by eq. 3. Storage and loss moduli vs. angular velocity (for the Maxwell model) are shown in Fig. 1a.

Stress relaxation time could be calculated from the condition: *G*^′^(*ω*^∗^) = *G*^"^(*ω*^∗^) (where ${\omega}^{\ast }=\frac{2\pi }{\tau_{R1}}$).

Various viscoelastic solid states are considered such as Kelvin-Voigt solid, transition state [17, 19] and jamming state [18] in the context of system ability to relax. The Kelvin-Voigt model equation is the simplest linear model suitable for describing viscoelastic solid behavior. It is expressed as:

4 $$ \sigma (t)={G}_s\varepsilon (t)+\eta \overset{.}{\varepsilon }(t) $$

where *G*_*s*_ is the elastic modulus. The strain relaxation under constant stress condition *σ*_0_ (i.e. creep experiments) could be expressed starting from the initial condition *ε*(*t* = 0) = 0 as: $\varepsilon (t)=\frac{\sigma_0}{G_s}\left(1-{e}^{-\frac{t}{\tau_{R2}}}\right)$ (where *ε*(*t*) is the strain and *τ*_*R*2_ is the strain relaxation time equal to ${\tau}_{R2}=\frac{\eta }{G_s}$). Strain increases from *ε*(*t* = 0) = 0 to *ε*(*∆t*). If the system undergoes free relaxation at *t* = *∆t* such that *σ* = 0 and *ε*(*∆t*) = *ε*_0_, the strain relaxation is expressed as $\varepsilon (t)={\varepsilon}_0{e}^{-t/{\tau}_{R2}}$. The Kelvin-Voigt model pointed out that stress cannot relax under constant strain conditions. The ability of a strain to relax is the main characteristic of viscoelastic solid. Complex modulus *G*^∗^(*ω*) could be formulated after Fourier transform of eq. 4. Corresponding storage and loss moduli could be expressed as:

5 $$ {G}^{\prime}\left(\omega \right)={G}_s\ {G}^{"}\left(\omega \right)=\upeta \upomega $$

Storage and loss moduli satisfy the following conditions (1) *G*^′^(*ω*)/*G*^"^(*ω*) > 1 at low angular velocities (solid-like behavior), (2) *G*^′^(*ω*)/*G*^"^(*ω*) < 1 at high angular velocities (liquid-like behavior), (3) *G*^′^(*ω*)~*G*_*s*_ and *G*^"^(*ω*)~*ω* at low and high angular velocities. Single strain relaxation time could be obtained on two ways: (1) by comparing experimental data of strain vs. time with model prediction obtained by eq. 4 or (2) by comparing experimental data of storage and loss moduli vs. angular velocity with model prediction obtained by eq. 5. Storage and loss moduli vs. angular velocity, for the Kelvin-Voigt model, are shown in Fig. 1b. Relaxation time for strain could be calculated from the condition: *G*^′^(*ω*^∗^) = *G*^"^(*ω*^∗^) (where ${\omega}^{\ast }=\frac{2\pi }{\tau_{R2}}$).

As was shown, viscoelastic liquid and viscoelastic solid account for both elastic and viscous behaviors. If *G*^′^(*ω*)/*G*^"^(*ω*) > 1, the elastic (solid-like) behavior is dominant, while if *G*^′^(*ω*)/*G*^"^(*ω*) < 1 the viscous (liquid-like) behavior is dominant. Maxwell and Kelvin-Voigt models point out to the presence of the single and finite relaxation times. In the case of the Maxwell model, it is the stress relaxation time. However, in the case of the Kelvin-Voigt model, it is the strain relaxation time. Despite the fact that both models account for the transition from a liquid-like to solid-like behavior and vice versa (Fig. 1a,b) none of them is able to describe the jamming state transition.

Tighe [17] and Braumgarten and Tighe [19] considered jamming state transition for the systems of dense packing soft, viscous, non-Brownian spheres. They distinguished three viscoelastic solid regimes: (1) the Kelvin-Voigt regime, (2) the transition regime, and (3) the jamming regime. System structural change within the transition regime is significantly damped which induces the anomalous nature of energy dissipation. The main indicator of this regime is the condition *G*^′^(*ω*) = *G*^"^(*ω*) [17]. The corresponding constitutive model for the transition regime could be expressed as:

6 $$ \sigma (t)={\upeta}_{\alpha }{D}^{\alpha}\left(\varepsilon (t)\right) $$

where η_*α*_ is the effective modulus, ${D}^{\alpha}\varepsilon (t)=\frac{d^{\alpha}\varepsilon (t)}{d{t}^{\alpha }}$ is the fractional derivative, and α is the order of fractional derivative (i.e. the damping coefficient of a system structural changes). Caputo’s definition of the fractional derivative of a function *ε*(*t*) was used and it is given as [31]:

7 $$ {D}^{\alpha}\varepsilon (t)=\frac{1}{\Gamma \left(1-\alpha \right)}\frac{d}{dt}{\int}_0^t\frac{\varepsilon \left({t}^{\prime}\right)}{{\left(t-t\prime \right)}^{\alpha }} dt^{\prime } $$

where Г (1 − *α*) is a gamma function. If the parameter is *α* = 0, we obtain *D*^0^*ε*(*t*) = *ε*(*t*). When *α* → 1, the corresponding fractional derivative is equal to ${D}^1\varepsilon (t)=\frac{d\varepsilon (t)}{d t}$. If the parameter *α* is equal to *α* = 0, the model eq. 6 represents reversible, elastic rheological behavior. However, if the parameter *α* is equal to *α* → 1, the model eq. 6 represents irreversible, viscous rheological behavior. Strain change under constant stress condition *σ*_0_ (i.e. creep experiments) can be expressed from eq. 6 as $\varepsilon (t)=\frac{\sigma_0}{\eta_{\alpha }}\frac{t^{\alpha }}{\varGamma \left(\alpha +1\right)}$. This result points out that strain cannot relax under constant stress condition. Strain increase depends on the model parameter *α*. We transformed eq. 6 from the time domain into the frequency domain using the Fourier integral transform. Fourier transform of the fractional derivative of the component of strain *ε*(*t*) is equal to *F*[*D*^*α*^(*ε*(*t*))] = (*iω*)^*α*^*F*[*ε*(*t*)]. The storage and loss moduli could be expressed as:

8 $$ {G}^{\prime}\left(\omega \right)={\upeta}_{\alpha }{\omega}^{\alpha}\mathit{\cos}\left(\frac{\pi \alpha}{2}\right)\ G"\left(\omega \right)={\upeta}_{\alpha }{\omega}^{\alpha}\mathit{\sin}\left(\frac{\pi \alpha}{2}\right) $$

Eq. 8 points out that the following condition for the transition regime *G*^′^(*ω*) = *G*^"^(*ω*) is satisfied for the damping coefficient equal to α = 1/2. This regime is established in the range of angular velocities *ω* ∈ [ *ω*_1_, *ω*_2_]. Systems cannot relax in this regime. Migration of the system constituents is strongly reduced within this regime.

Honter and Weeks [18] characterized rheologically the jamming/glass state of colloidal systems such that (1) the diffusion coefficient of the system constituents D →0, (2) the system relaxation time is *τ*_*R*_ → ∞, and (3) the viscoelastic solid condition should be satisfied, i.e. *G*^′^(*ω*)/*G*^"^(*ω*) > 1. The infinity of the relaxation time indicates the condition that storage and loss moduli are parallel, i.e. *G*^′^(*ω*)/*G*^"^(*ω*) = *const* > 1. This result points that model eq. 6 could be applied for describing the jamming/glass state such that the damping coefficient should be less than ½ and equal to 0 < *α* < 1/2. However, if the damping parameter is *α* = 0, the relaxation time also becomes equal to zero, i.e. *τ*_*R*_ = 0, while a dissipative phenomenon disappears, i.e. *G* " (*ω*) = 0. In this case, the rheological behavior is reversible, elastic. This result points out that relaxation time represents the key parameter for determining the jamming state. Strain change under constant stress condition *σ*_0_ for the initial condition *ε*(0) = 0 is equal to $\varepsilon (t)=\frac{\sigma_0}{\eta_{\alpha }}\frac{t^{\alpha }}{\varGamma \left(\alpha +1\right)}$ (where *Г*(*α* + 1) is a gamma function). Strain increases during the time period *∆t* from *ε*(0) = 0 to *ε*(*∆t*) without the ability to relax. If the system undergoes free relaxation at *t* = *∆t* such that *σ* = 0 and *ε*(*∆t*) = *ε*_0_, the strain stays constant for *t* > *∆t* and equal to *ε*(*t*) = *ε*_0_. Consequently, a strain cannot relax under constant stress condition similarly as for viscoelastic liquid described by eq. 1.

### Viscoelastic regimes

**Fig. 2:**
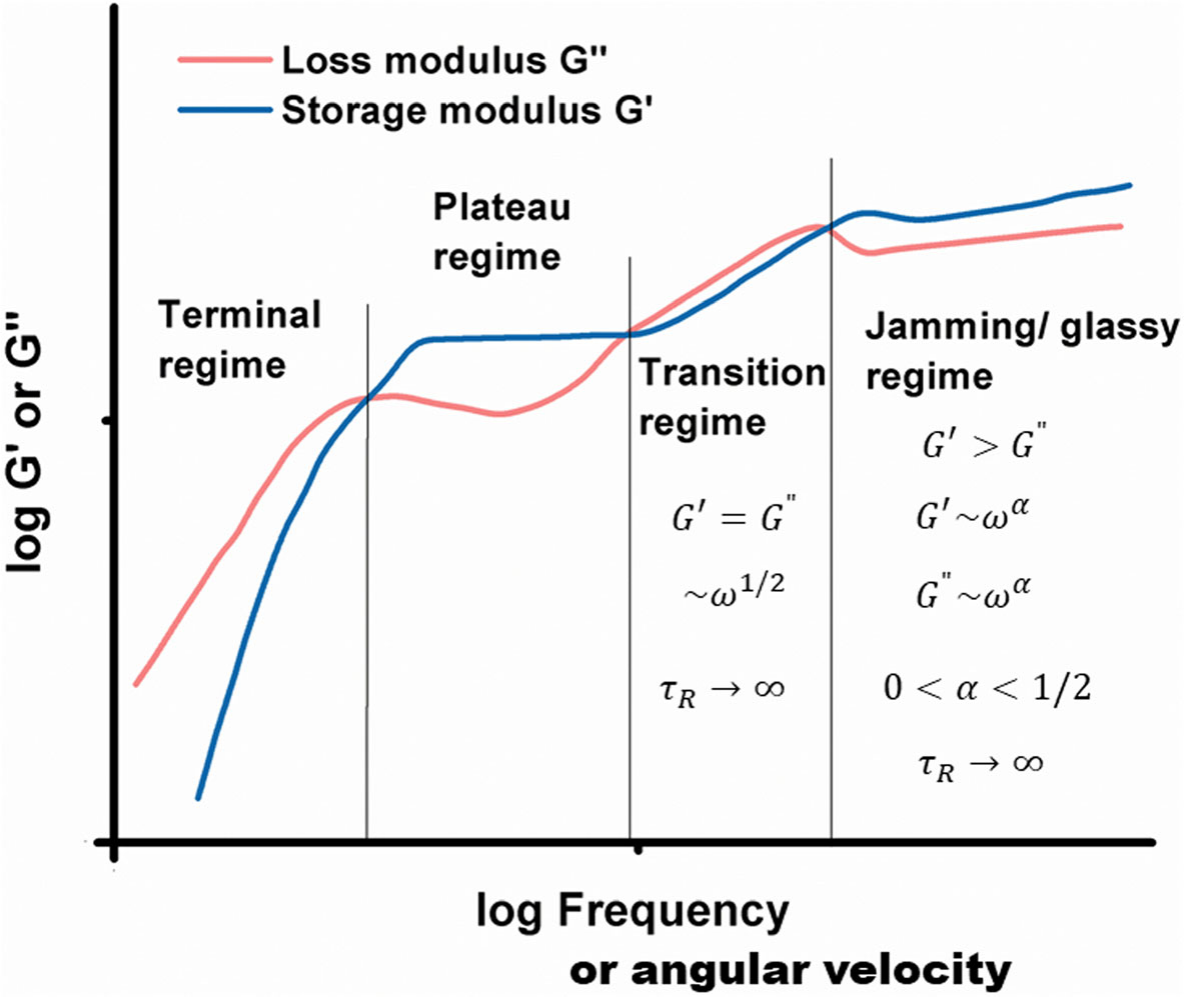
Schematic presentation of storage and loss moduli vs. angular velocity for: (1) the terminal regime (the Maxwell model, eq. 3), (2) the plateau regime (the Kelvin-Voigt model, eq. 5), (3) the transition regime (eq. 8 for the damping coefficient equal to α = 1/2), and (4) the jamming/glass regime (eq. 8 for the damping coefficient in the range of 0 < *α* < 1/2)

All described types of viscoelastic behavior such as: (1) the Maxwell viscoelastic liquid regime, (2) the Kelvin-Voigt viscoelastic solid regime, (3) the transition regime, and (4) the jamming regime can be presented together schematically in the form of storage and loss moduli vs. angular velocity (Fig. 2).

**Fig. 3:**
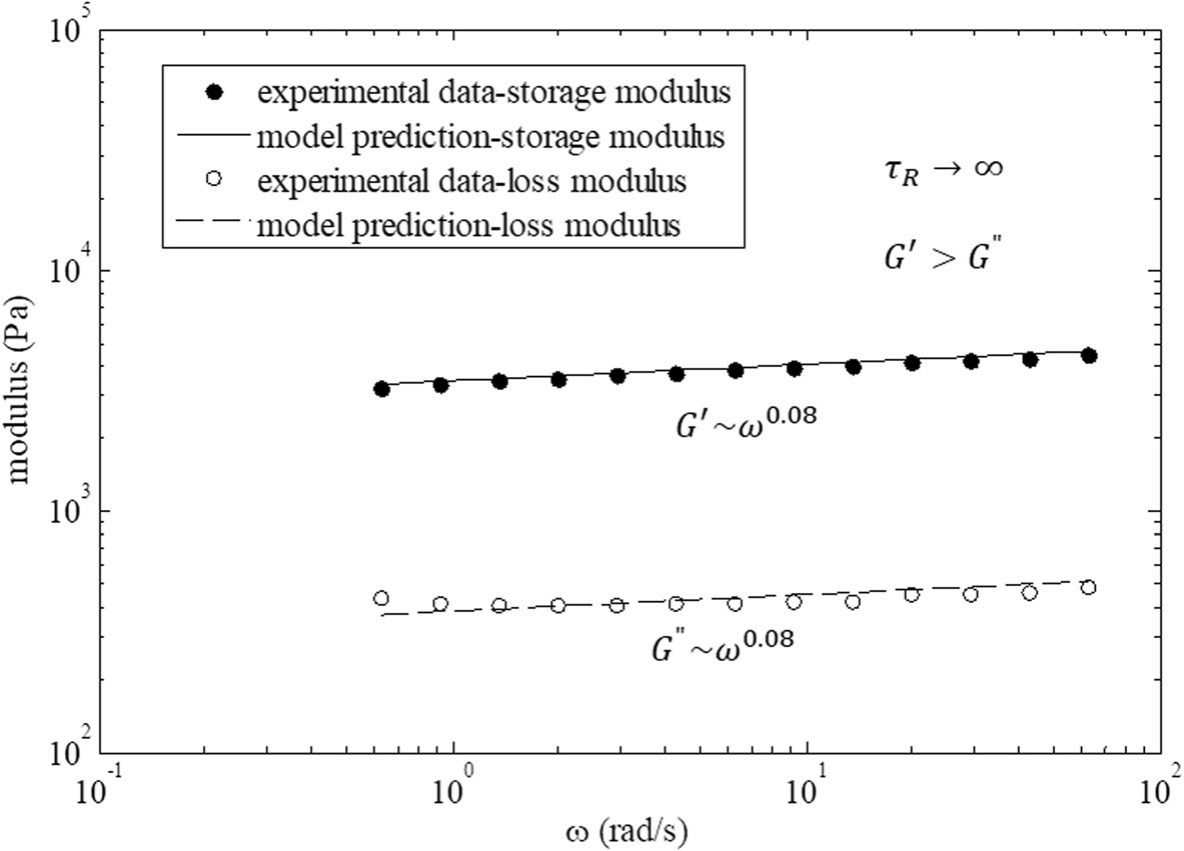
Storage and loss moduli vs. angular velocity for a monolayer of Ca-alginate beads under jamming state calculated by eq. 8 for the model parameters: the damping coefficient equal to *α* = 0.08 and the effective modulus equal to η_*α*_ = (3.4 ± 0.1)*x*10^3^ *Pas*^*α*^, obtained by Pajic-Lijakovic et al. [32]

Liu et al. [20] obtained a similar result by considering the rheological response of coacervate-based systems. Braumgarten and Tighe [19] considered jamming state transition for the systems of dense packing soft, viscous, non-Brownian spheres. They distinguished (1) the Kelvin-Voigt viscoelastic solid regime, (2) the transition regime, and (3) the jamming regime. Pajic-Lijakovic et al. [32] considered viscoelasticity of the monolayer made by dense packed Ca-alginate beads (average diameter is 2 *mm*). A rheological response of this monolayer under low oscillator strain condition corresponds to jamming state. One experimental set in the context of storage and loss moduli vs. angular velocity is shown in Fig. 3.

Storage and loss moduli satisfy the condition that *G*^′^(*ω*)/*G*^"^(*ω*) = *const* > 1, while the corresponding damping coefficient is equal to *α* = 0.08 and the effective modulus is η_*α*_ = (3.4 ± 0.1)*x*10^3^ *Pas*^*α*^. Constitutive model eq. 8 is suitable for describing this rheological behavior. We would like to consider the viscoelasticity of multicellular systems caused by collective cell migration in the context of the jamming state transition.

## Results

The consideration of the long-time viscoelasticity of multicellular systems caused by collective cell migration is a difficult task due to the fact that only a few examples have been elaborated properly in the literature from the rheological standpoint. One of them is cell aggregate uni-axial compression between parallel plates. Stress relaxation under constant aggregate shape condition, cell aggregate shape relaxation under constant stress condition or free relaxation after stress action has been considered by Mombash et al. [33] and Marmottant et al. [29]. We introduced the following features of cell rearrangement in the context of multicellular system ability to relax:

1. Marmottant et al. [29] considered stress relaxation under the constant aggregate shape. They proposed a single relaxation time for stress equal to a few minutes. Stress relaxes exponentially from ~27 *Pa* to the residual stress value equal to ~17 *Pa* during 25 *min*. The phenomenon of stress relaxation could be related to the adaptation of adhesion contacts [2].
2. Marmottant et al. [29] also considered the aggregates free relaxation (its rounding) after uni-axial compression during 1 h. They reported that the aggregate shape first relaxes quickly (the relaxation time corresponds to a few minutes) and then more slowly (the relaxation time corresponds to tens of minutes). Consequently, the shape relaxation is accomplished via two mechanisms related to (1) structural changes of adhesion complexes (which leads to the cell packing density relaxation) obtained at minute scale and (2) collective cell migration obtained at hour time scale [29, 34].
3. Mombash et al. [33] pointed to one average relaxation time (obtained at hour scale) for the aggregate shape relaxation by collective cell migration (its rounding). Pajic-Lijakovic et al. [25] pointed out that aggregate shape and surface relaxation represent a characteristic of viscoelastic solid rather than viscoelastic liquid.
4. Two constitutive models suitable for viscoelastic solid can relate stress and the aggregate shape parameter *ε*_*d*_(*t*) = *AR*(*t*) − 1 [33] (where *AR*(*t*) is the aggregate aspect ratio). One is Zener model while the other is the Four-parameter model. Zener model introduces one relaxation time for stress and the other for the aggregate shape parameter which corresponds to experimental results by Mombash et al. [33]. Four-parameter model introduces one relaxation time for stress and two relaxation times for the aggregate shape parameter which corresponds to the experimental results from Marmottant et al. [29]. Zener model is expressed as:

9 $$ \sigma (t)+{\tau}_{R1}\frac{d\sigma (t)}{d t}={G}_s{\varepsilon}_d(t)+\upeta \frac{d{\varepsilon}_d(t)}{d t} $$

where *τ*_*R*1_ is the relaxation time for stress while the relaxation time for the aggregate shape parameter is equal to ${\tau}_{R2}=\frac{\upeta}{G_s}$. Stress relaxation under constant aggregate shape *ε*_*d*0_, for the initial condition *σ*(*t* = 0) = 0, is equal to $\sigma (t)={\sigma}_R\left(1-{e}^{-\frac{t}{\tau_{R1}}}\right)$ (where *σ*_*R*_ is the residual stress equal to *σ*_*R*_ = *G*_*s*_*ε*_*d*0_). Aggregate shape relaxation under constant stress *σ*_0_, for the initial condition *ε*_*d*_(*t* = 0) = 0, is equal to ${\varepsilon}_d(t)=\frac{\sigma_0}{G_s}\left(1-{e}^{-\frac{t}{\tau_{R2}}}\right)$. The Four-parameter model is expressed as: $\sigma (t)+{\tau}_{R1}\frac{d\sigma (t)}{d t}={G}_s{\varepsilon}_d(t)+\upeta \frac{d{\varepsilon}_d(t)}{d t}+\upeta {t}_R\frac{d^2{\varepsilon}_d(t)}{d{t}^2}$ (where *t*_*R*_ is the characteristic time for the shape parameter change). Stress relaxation under constant shape parameter *ε*_*d*0_ is similar as for the Zener model. The aggregate shape relaxation under constant stress condition *σ*_0_ (i.e. creep experiments) can be expressed starting from the initial conditions *ε*_*d*_(*t* = 0) = 0 and $\frac{d{\varepsilon}_d\left(t=0\right)}{dt}={\overset{.}{\varepsilon}}_{d0}$ as: ${\varepsilon}_d(t)={C}_1{e}^{-\frac{t}{\tau_{R1}^{\ast }}}+{C}_2{e}^{-\frac{t}{\tau_{R2}^{\ast }}}+\frac{\sigma_0}{G_s}$ (where ${C}_1=\frac{{\overset{.}{\varepsilon}}_{d0}-\frac{\sigma_0}{G_s\ }\frac{1}{\tau_{R1}^{\ast }}}{\frac{1}{\tau_{R2}^{\ast }}-\frac{1}{\tau_{R1}^{\ast }}}$, ${C}_2=\frac{{\overset{.}{\varepsilon}}_{d0}-\frac{\sigma_0}{G_s\ }\frac{1}{\tau_{R2}^{\ast }}}{\frac{1}{\tau_{R2}^{\ast }}-\frac{1}{\tau_{R1}^{\ast }}}$ are constants, while ${\tau}_{R1}^{\ast }=\frac{2\frac{\upeta}{G_s}{t}_R}{\frac{\upeta}{G_s}+\sqrt{{\left(\frac{\upeta}{G_s}\right)}^2-4\frac{\upeta}{G_s}{t}_R}}$, and ${\tau}_{R2}^{\ast }=\frac{2\frac{\upeta}{G_s}{t}_R}{\frac{\upeta}{G_s}-\sqrt{{\left(\frac{\upeta}{G_s}\right)}^2-4\frac{\upeta}{G_s}{t}_R}}$ are the short and long relaxation times for the aggregate shape parameter.

**Fig. 4:**
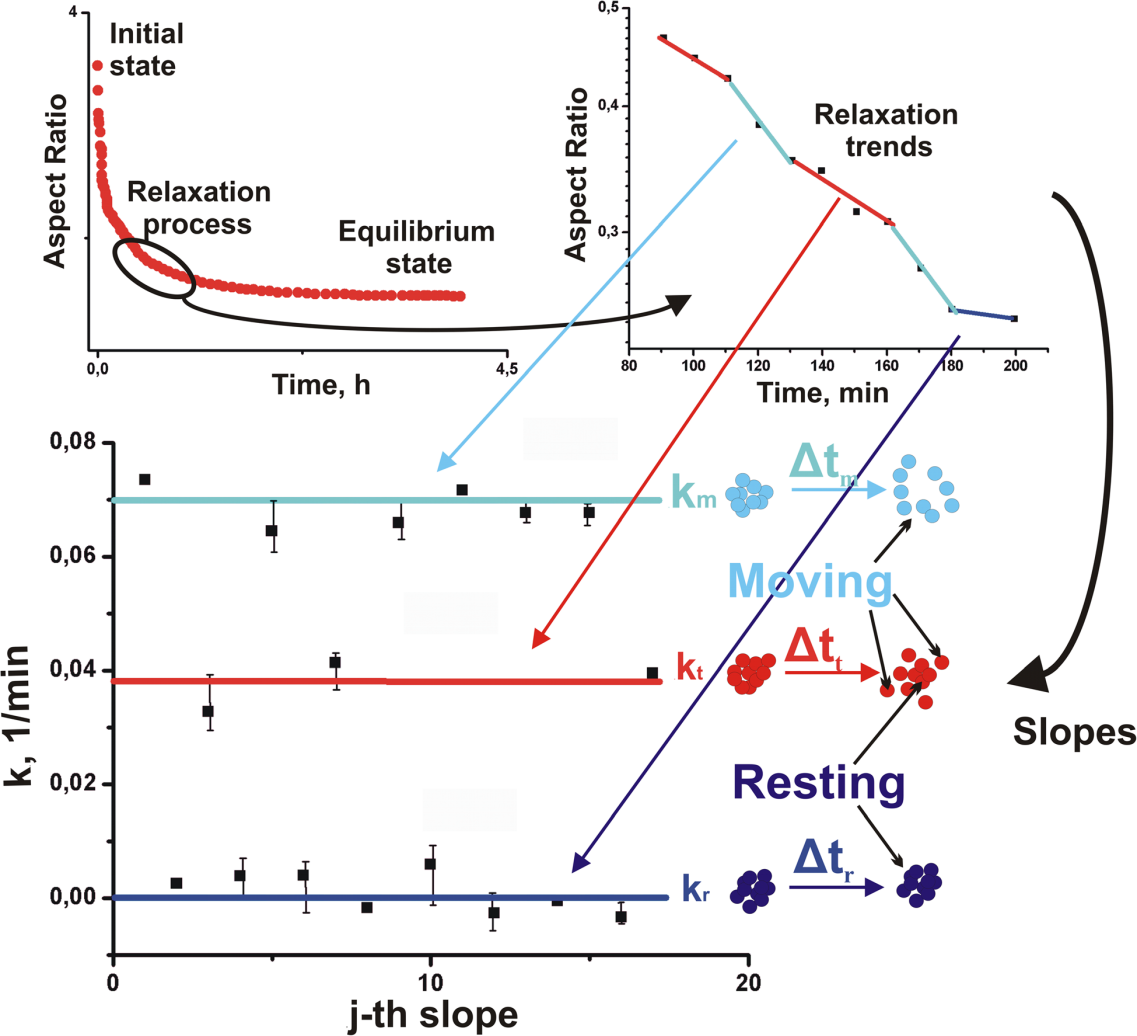
Aggregate aspect ratio vs. time in normal and log-normal forms were calculated from the experimental data for 3D chicken cell aggregate rounding after uniaxial compression obtained by Schotz et al. [35] for 3D zebrafish ectodermal aggregate and elaborated by Pajic-Lijakovic and Milivojevic [25]

1. Pajic-Lijakovic and Milivojevic [25] considered experimental data by Mombash et al. [33]. They pointed out that the aggregate shape free relaxation occurs via successive relaxation cycles. The relaxation rates *k*^*j*^ (where *k*^*j*^ is the relaxation rate for the *j*-^th^ cycle) are not random but gather around two or three values indicating various scenarios of cell migration. Three scenarios of cell migration were discussed as (1) *k*_*m*_- the most of cells migrate, (2) *k*_*r*_ ≪ *k*_*m*_ - the most of cells stay in the resting state and in some cases *k*_*r*_ ≈ 0, and (3) *k*_*r*_ < *k*_*t*_ < *k*_*m*_- some cell groups migrate while the others, at the same time, stay in resting state. Aggregate shape relaxation in the form of the aggregate aspect ratio vs. time for the experimental data by Mombash et al. [33] is shown in normal and log-normal forms (Fig. 4).

The normal form is suitable to present initial and relaxed (equilibrium) state of the aggregate. The log-normal form is suitable to present successive relaxation cycles during aggregate rounding.

Change of the relaxation rate from cycle to cycle is induced by uncorrelated motility. Uncorrelated motility represents the consequence of time delay in cell response to a various mechanical and biochemical stimulus caused by gene expression [36]. This time delaying might be relevant for cell coupling because what cells acquire at the present time is the information of surrounding cells some time ago. These perturbations can induce that (1) cells in the same population respond to different signals and/or (2) cells behave differently in response to the same signals [37, 38]. These phenomena can lead to the collision of velocity fronts [13, 14, 25, 27] and also to changes in the configuration of migrating cells from cycle to cycle [25]. Nnetu et al. [28] considered the collision of velocity fronts in 2D in the context of jamming state.

### Cell aggregate compression: volumetric and surface viscoelasticity

External uni-axial stress ${\sigma}_e=\frac{F}{S}$ (where *F* is the force act on the plate and *S* is the surface contact area between the plate and the aggregate) induces the generation of internal stress *σ* within the aggregate such that *σ*_*e*_ = *σ* = *const*. We consider aggregate shape change under constant stress condition and free relaxation after compression in the context of the jamming state proposed by eq. 6 in order to recognize the appearance of this state in experiments. Aggregate internal stress *σ* represents a product of surface viscoelasticity and volumetric viscoelasticity and could be expressed by Young-Laplace law. We modified the Young-Laplace law proposed by Marmottant et al. [29] in order to account for observed successive relaxation cycles. Consequently, the internal stress for the j-^th^ cycle represents the consequence of surface and volumetric effects and can be expressed as:

10 $$ \sigma ={\Delta  p}^j+{\tau}^j $$

where *τ*^*j*^ is the volumetric contribution to stress, *∆p*^*j*^ is the hydrostatic pressure during the j-^th^ relaxation cycle that is equilibrated with the corresponding value of the tissue surface tension *γ*^*j*^ such that *∆p*^*j*^ = *γ*^*j*^*H*^*j*^, *H*^*j*^ (the Young-Laplace law) is the corresponding aggregate curvature. Volumetric viscoelasticity is quantified by the stress *τ*^*j*^ while the surface viscoelasticity quantified by dynamic surface tension *γ*^*j*^. Mombash et al. [33] and Pajic-Lijakovic and Milivojevic [25] pointed out to the time change of tissue surface tension during aggregate rounding after uni-axial compression. Cell aggregate rounding is discussed in the context of jamming state transition based on the proposed model by Pajic-Lijakovic and Milivojevic [25].

## Discussion

Three scenarios of cell migration established during aggregate rounding have been discussed based on experimental data by Mombash et al. [33]. The free relaxation satisfies the condition *σ* = 0. For this condition, the tissue surface tension and viscoelastic contribution to stress, expressed by eq. 10, could be related as *∆γ*^*j*^*H*^*j*^ = *∆τ*^*j*^ (where *∆γ*(*t*) = *γ*(*t*) − *γ*_0_ is the tissue surface tension difference and *γ*_0_ is the equilibrium value of the surface tension). This important result points out to inter-relation between the volumetric viscoelasticity and the surface viscoelasticity.

### Scenarios of cell migration

Cell rearrangement was treated as T1 process in the form of the Eyring model proposed by Marmottant et al. [29]. Energy barrier *∆E*_*T*1_ influences the cell state transition from migrating to resting *m* → *r* and vice versa *r* → *m* expressed as [25]:

11 $$ \frac{d\Delta  {\gamma}_r(t)}{dt}=-{\lambda}_{r\to m}\Delta  {\gamma}_r(t)+{\lambda}_{m\to r}\Delta  {\gamma}_m(t) $$

where *∆γ*_*r*_(*t*) is the dynamic surface tension contribution from resting cells equal to *∆γ*_*r*_(*t*) = *γ*_*r*_(*t*) − *γ*_0_ and *∆γ*_*m*_(*t*) is the dynamic surface tension contribution from migrating cell groups equal to *∆γ*_*m*_(*t*) = *γ*_*m*_(*t*) − *γ*_0_. Total dynamic surface tension represents the sum of contributions from migrating and from resting cells, i.e. *∆γ*(*t*) = *∆γ*_*m*_(*t*) + *∆γ*_*r*_(*t*). The specific rate for *r* → *m* transition *λ*_*r* → *m*_ is expressed as ${\lambda}_{r\to m}=\lambda {e}^{-\frac{\Delta  {E}_{T1}-\Delta  {E}_{eff}}{k_B{T}_{eff}}}$, λ is the characteristic frequency, *k*_*B*_ is the Boltzmann constant, *T*_*eff*_ is the effective temperature. Concept of effective temperature has been applied for considering rearrangement of various thermodynamical systems (near to equilibrium and far from equilibrium) from glasses and sheared fluids to granular systems [39]. Pajic-Lijakovic and Milivojevic [9] applied this concept to cell long-time rearrangement of dense cellular systems. The effective temperature, in this case, represents a product of cell migration and is expressed based on a generalization of Einstein’s relation [39] as ${k}_B{T}_{eff}=\frac{D}{\mu^{\prime }}$, (where *D* is the diffusivity of migrating cells and *μ*′ is the mobility of velocity fronts). The energetic barrier for cell long-time rearrangement *∆E*_*T*1_ represents a strain energy threshold. For the strain energy *W* = *σδε*_*d*_*∆V* larger than the energy barrier *∆E*_*T*1_ collective cell migration occurs (where *σ* is the internal stress, *∆V* is the volumetric change of the aggregate surface region). This cell rearrangement represents a result of cell tendency to decrease strain energy. The specific rate for *m* → *r* transition *λ*_*m* → *r*_ is expressed as ${\lambda}_{m\to r}=\lambda {e}^{-\frac{\Delta  {E}_{T1}+\Delta  {E}_{eff}}{k_B{T}_{eff}}}$. The effective driving energy is equal to *∆E*_*eff*_ = *γ*_0_*∆A* − *∆E*_*p*_ (where *∆A* is the aggregate surface change during the aggregate rounding, *∆E*_*p*_ is the energy perturbations caused by uncorrelated motility). The energy perturbations *∆E*_*p*_ accounts for cumulative effects of mechanical and biochemical perturbations which lead to a collision of velocity fronts and stagnant zones formation. Stagnant zones represent a local increase of cells in the resting state. The ratio $\frac{\lambda_{r\to m}}{\lambda_{m\to r}}$ is equal to $\frac{\lambda_{r\to m}}{\lambda_{m\to r}}={e}^{-\frac{2\Delta  {E}_{eff}}{k_B{T}_{eff}}}$. Three causes were established depending on the energy perturbation *∆E*_*p*_:

1. *γ*_0_∆*A* ≫ ∆*E*_*p*_ which corresponds to ${\lambda}_{r\to m}={\lambda}_{m\to r}\ {e}^{-\frac{2{\gamma}_0\Delta  A}{k_B{T}_{eff}}}$;
2. *γ*_0_∆*A*~∆*E*_*p*_ which corresponds to *λ*_*r* → *m*_~*λ*_*m* → *r*_, and
3. *γ*_0_∆*A* ≪ ∆*E*_*p*_ which corresponds to *λ*_*r* → *m*_ ≪ *λ*_*m* → *r*_.

Consequently, biochemical and mechanical energy perturbations represent the key parameter for establishing various scenarios of migrating cells. We supposed that contribution of resting cells to the dynamic surface tension is reversible while the contribution of migrating cells is dissipative and proved this statement by comparing experimental data obtained by Mombash et al. [33] with the model prediction. The contribution of resting cells could be expressed as [25]:

12 $$ \Delta  {\gamma}_r(t)={E}_{app}{\varepsilon}_d(t) $$

where *E*_*app*_ is the apparent surface elasticity modulus. The contribution of migrating cells could be expressed as

13 $$ \Delta  {\gamma}_m(t)={\eta}_{app}\frac{d{\varepsilon}_d(t)}{dt} $$

where *η*_*app*_ is the apparent surface viscosity. Changes in the deformation parameter with time could be expressed by introducing eqs. 12 and 13 into eq. 11 as:

14 $$ \frac{d{\varepsilon}_d(t)}{dt}+k{\varepsilon}_d(t)=0 $$

where $k=\frac{\lambda_{r\to m}\ {E}_{app}}{E_{app}-{\lambda}_{m\to r}{\eta}_{app}}$ is the aggregate shape relaxation rate. The relaxation rate is equal to:

1. ${k}_m=\frac{\lambda_{m\to r}\ {e}^{-\frac{2{\gamma}_0\Delta  A}{k_B{T}_{eff}}}{E}_{app}}{E_{app}-{\lambda}_{m\to r}{\eta}_{app}}$ for the case 1,
2. ${k}_t={k}_m\ {e}^{-\frac{2{\gamma}_0\Delta  A}{k_B{T}_{eff}}}$ for the case 2, and
3. *k*_*r*_ → 0 for the case 3 as schematically presented in Fig. 4.

The aggregate surface energy *γ*_0_*∆A* relative to a specific energy of collectively migrated cells *k*_*B*_*T*_*eff*_ can be estimated from the experimentally determined ratio of the relaxation rates $\frac{k_m}{k_t}$. Pajic-Lijakovic and Milivojevic [25] calculated the ratio $\frac{k_m}{k_t}$ from the experimental data by Mombach et al. [33] and Schotz et al. [35]. Mombash et al. [33] examined the aggregate rounding for 3D chicken embryonic neural retina aggregates with various radius: *R* = 87 *μm*, and *R* = 65 *μm*. The corresponding energetic ratio $\frac{\gamma_0\Delta  A}{k_B{T}_{eff}}$ is equal to $\frac{\gamma_0\Delta  A}{k_B{T}_{eff}}=0.36\pm 0.04$. Schotz et al. [35] examined the aggregate rounding for 3D zebrafish ectodermal aggregates. The corresponding energetic ratio $\frac{\gamma_0\Delta  A}{k_B{T}_{eff}}$ is equal to $\frac{\gamma_0\Delta  A}{k_B{T}_{eff}}=0.33\pm 0.02$.

Case 1 corresponds to an ordered trend of cell migration characterized by a minimum of energy perturbations. The relaxation time for the aggregate shape is finite and equal ${\tau}_R={k}_m^{-1}$ which indicates viscoelastic solid.

Case 2 corresponds to a disordered trend of cell migration characterized by a medium value of the energy perturbations. The relaxation time for the aggregate shape is finite and equal ${\tau}_R={k}_t^{-1}$ which also indicates viscoelastic solid.

Case 3 corresponds to significantly suppressed cell migration caused by large energy perturbations. The relaxation time is ${\tau}_R={k}_r^{-1}\to \infty$ while the aggregate shape parameter is *ε*_*d*_(*t*) ≈ *const*. If case 3 corresponds to viscoelastic solid these conditions point out to the jamming state described by eq. 6. Viscoelastic liquid described by the Maxwell model (eq. 1) also satisfies the conditions that for *σ* = 0, the surface strain (or the shape deformation parameter) is constant, i.e. *ε*_*d*_(*t*) ≈ *const*, but it is not the jamming state.

Based on experimental data by Mombash et al. [33] jamming state frequently occurred during the aggregate rounding. Cells are able to overcome this undesirable state and start moving again in the next cycle. A similar trend has been observed in 2D dynamics. Nnetu et al. [28] considered the state of the boundary layer formed after a collision of velocity fronts. They pointed out that collision turns cells within the boundary in a jamming state. After collision stress locally increases without the ability to relax. This stress induces the generation of a compressive strain which can lead to an increase of cell packing density. This increase is much damped for the jamming state in comparison with other solid states. As time increases, marginal cells start moving and this movement unjams the boundary.

Experimental data for the aggregate shape relaxation after uni-axial compression shows a relaxation trend in the form of successive relaxation cycles. Model eq. 14 should be expressed for every cycle for *t* ∈ [0, *∆t*^*j*^] as $\frac{d{\varepsilon}_d{(t)}^j}{dt}+{k}^j{\varepsilon}_d{(t)}^j=0$ (where *k*^*j*^ is the relaxation rate for the j-^th^ cycle which can be equal to *k*_*m*_, *k*_*t*_, or *k*_*r*_ and *∆t*^*j*^ is the time period for j-^th^ cycle). The total time period for the aggregate rounding is equal to $\Delta  {t}_T=\sum \limits_{j=1}^n\Delta  {t}^j$. Experimental data by Mombash et al. [33] pointed that a total number of relaxation cycles varies from 7 to 15 depending on cell types and experimental conditions. Model eq. for j-^th^ cycle is solved starting from the initial condition at *t* = 0 the aggregate shape parameter is equal to ${\varepsilon}_d{\left(t=0\right)}^j={\varepsilon}_{d0}^j$. Accordingly, the aggregate shape relaxation for the *j*-^th^ cycle could be expressed as:

15 $$ {\varepsilon}_d{(t)}^j={\varepsilon}_{d0}^j\ {e}^{-{k}^jt} $$

where *ε*_*d*_(*t*)^*j*^ is the aggregate deformation parameter during the j-^th^ relaxation cycle, ${\varepsilon}_{d0}^j$ is the initial value of the deformation parameter, and *k*^*j*^ is the relaxation rate for the *j*-^th^ cycle. Model eq. 15 satisfies the functional trend of the experimental data by Mombash et al. [33]. Mechanical and biochemical perturbations *∆E*_*p*_, as key factors which can induce jamming state transition during aggregate rounding, depends on cell type, a magnitude of applied stress and loading time.

## Conclusion

The main goal of this theoretical consideration is to (1) describe jamming state from the rheological point of view and (2) consider long-time cell rearrangement caused by collective cell migration based on formulated jamming state transition. Jamming state represents particular non-linear viscoelastic solid state characterized by the following conditions: (1) the migration of the system constituents is significantly damped such as the diffusion coefficient tends to zero, i.e. *D* → 0, (2) the relaxation time tends to infinity, i.e. *τ*_*R*_ → ∞, (3) systems structural changes induce the anomalous nature of energy dissipation that can be modelled by introducing the fractional derivatives, (4) the order of fractional derivative (the damping coefficient) should be equal to 0 < *α* < 1/2, and (5) storage modulus is higher than loss modulus, and satisfies the condition *G*^′^(*ω*)/*G*^"^(*ω*) = *const* > 1. Consequently, a system trapped in a jamming state cannot relax. The main parameter for the jamming state should be a relaxation time. Single finite values of the relaxation time are obtained for viscoelastic liquid described by the Maxwell model and viscoelastic solid described by the Kelvin-Voigt model. For elastic solid, the relaxation time tends to zero. However, for the jamming state, the relaxation time tends to infinity.

We considered cell long-time rearrangement via collective cell migration during aggregate rounding after uni-axial compression in the context of jamming state transition. Aggregate shape relaxes exponentially during successive relaxation cycles. Change of the relaxation rate from cycle to cycle is induced by uncorrelated motility. Uncorrelated motility represents the consequence of mechanical and biochemical perturbations *∆E*_*p*_. These perturbations can induce the collision of velocity fronts which lead to change in the configuration of migrating cells from cycle to cycle. Three scenarios of cell migration were considered: (1) most of the cells migrate (*∆E*_*p*_ is minimal), (2) some cell groups migrate while the others (at the same time) stay in resting state (*∆E*_*p*_ is medium), and (3) most of the cells are in resting state (*∆E*_*p*_ is maximal). The third scenario corresponds to viscolelastic solid under the jamming state. If the cells reach the jamming state in one cycle by uncorrelated motility, cells are able to overcome this undesirable state and start migrating again in the next cycle by achieving the first or second scenarios. This spontaneous un-jamming is a unique characteristic of living systems and is induced by the cumulative effects of biochemical processes such as cell signaling and gene expression.

## Data Availability

Not applicable.
